# Possible combined effects of climate change, deforestation, and harvesting on the epiphyte *Catopsis compacta*: a multidisciplinary approach

**DOI:** 10.1002/ece3.765

**Published:** 2013-09-17

**Authors:** Rafael F del Castillo, Sonia Trujillo-Argueta, Raul Rivera-García, Zaneli Gómez-Ocampo, Demetria Mondragón-Chaparro

**Affiliations:** 1 Instituto Politécnico Nacional, CIIDIR OaxacaHornos 1003, Santa Cruz Xoxocotlan, Oaxaca, 71239, Mexico; 2 Yale University, Yale Peabody Museum of Natural History170 Whitney Avenue, New Haven, Connecticut, 06520, USA

**Keywords:** Altitudinal gradient, demography, edge effects, fragmentation, genetic diversity, harvesting, nontimber forest products, plant cover dynamics.

## Abstract

Climate change, habitat loss, and harvesting are potential drivers of species extinction. These factors are unlikely to act on isolation, but their combined effects are poorly understood. We explored these effects in *Catopsis compacta*, an epiphytic bromeliad commercially harvested in Oaxaca, Mexico. We analyzed local climate change projections, the dynamics of the vegetation patches, the distribution of *Catopsis* in the patches, together with population genetics and demographic information. A drying and warming climate trend projected by most climate change models may contribute to explain the poor forest regeneration. *Catopsis* shows a positive mean stochastic population growth. A PVA reveals that quasi-extinction probabilities are not significantly affected by the current levels of harvesting or by a high drop in the frequency of wet years (2%) but increase sharply when harvesting intensity duplicates. Genetic analyses show a high population genetic diversity, and no evidences of population subdivision or a past bottleneck. Colonization mostly takes place on hosts at the edges of the fragments. Over the last 27 years, the vegetation cover has being lost at a 0.028 years^−1^ rate, but fragment perimeter has increased 0.076 years^−1^. The increases in fragment perimeter and vegetation openness, likely caused by climate change and logging, appear to increase the habitat of *Catopsis,* enhance gene flow, and maintain a growing and highly genetically diverse population, in spite of harvesting. Our study evidences conflicting requirements between the epiphytes and their hosts and antagonistic effects of climate change and fragmentation with harvesting on a species that can exploit open spaces in the forest. A full understanding of the consequences of potential threatening factors on species persistence or extinction requires the inspection of the interactions of these factors among each other and their effects on both the focus species and the species on which this species depends.

## Introduction

Climate change, land-use change, and harvesting are drivers of species extinction of global importance (Foley et al. 2005; Beever and Belant 2012). Therefore, understanding the effects of these factors is essential for the design of conservation, sustainable management, and restoration plans. In many cases, these factors act simultaneously on the species, but their combined effects need to be investigated. The little evidence available reveals that a common species response to the combined effects of such factors is unlikely. For instance, habitat degradation and climate change are likely to decrease habitat specialists and benefit some mobile and generalist species of British butterflies (Warren et al. 2001). For exploited species, harvesting effects adds a further complication, which combined effects with other potential drivers of species extinction are yet to be explored.

Epiphytes comprise 10% of all vascular plants, and some epiphyte species are among the most vulnerable organisms to climate change, harvesting, and disturbance (Benzing 1998; Ozanne et al. 2003). Climate change may affect epiphytes directly and indirectly. As some of these plants obtain nutrients and water from the atmosphere, changes in climate, in particular in temperature and humidity, affect their water and nutrient supplies (Obregona et al. 2011). Climate change affects epiphytes indirectly by affecting the host plants on which they live (Hsu et al. 2012). Global warming, for instance, is expected to promote uphill displacements and range contractions of populations inhabiting mountain ranges (Wilson and Gutiérrez 2012). Such changes may benefit the epiphytes at certain altitudes and harm at others. The hosts are likely to be affected as well, although not necessarily with the same effects observed in the epiphytes.

Land-use change usually reduces the abundance of host trees and, consequently, is expected to decrease the abundance and diversity of epiphytes. Disturbances associated with land-use change frequently result in higher solar radiation, temperature and wind velocity, and lower humidity compared with the original conditions, resulting in a gradual substitution of mesic epiphytes by more xerophytic epiphytes (Cascante-Marín et al. 2006). Epiphyte harvesting is very common in certain geographical regions (Flores-Palacios and Valencia Díaz 2007), but its consequences are poorly known. Harvesting may endanger some populations (Borba et al. 2007). However, harvesting effects may depend on human culture and taxonomic groups and may allow a higher degree of biodiversity conservation, compared with other land-use alternatives, such as the conversion of forest to pastures (Ruiz-Pérez 1997).

Assessing the mechanisms underlying the impacts of human activities on epiphytes such as harvesting requires the inclusion of multiple ecological levels ranging from genes to ecosystems (Ticktin 2004). Population genetic studies are useful to detect the genetic diversity of populations. Theory predicts that the capability of populations to respond to environmental changes depends on their genetic diversity. This appears to be the case for climate change in aquatic plant species (Ehlers and Reusch 2008), and for fragmentation in tropical tree species (del Castillo et al. 2011). Human activities have yielded conflicting results in epiphyte genetic diversity. Harvesting has been associated with decreases in genetic diversity (Cruse-Sanders and Hamrick 2004), but some epiphytes maintain considerable levels of genetic diversity in fragmented habitats (Avila Diaz and Oyama 2007). In addition to estimating genetic diversity, population genetic analyses allow exploring the effects of fragmentation on gene flow (Murren 2003), and detecting past bottlenecks (Alcántara et al. 2006), processes that may affect the population's abundance and persistence. Demographic studies are useful for assessing the effects of anthropogenic factors on populations. Harvesting, for instance, is one source of mortality, which affects the population vital rates and may increase the extinction risks in epiphytes (Mondragón 2009; Mondragón and Ticktin 2011). Finally, the consideration of the epiphyte ecosystems and its dynamics may help to interpret the observed population characteristics of epiphytes in ecosystems affected by anthropogenic activities. Forest dynamics, in particular, is expected to play a crucial role in epiphyte abundance as shown in simulation models (e.g., Hsu et al. 2012). Vegetation dynamics usually alter the distribution of epiphytes' microhabitats (Zartman 2003).

Despite their importance, comprehensive studies on epiphytes that include population genetics, demography, and the dynamics of the specific portions of the landscape at which the studied population dwells are lacking. As part of a long-term project aiming at fomenting the conservation of native forests and their species in southern Mexico, we conducted a multidisciplinary study on *Catopsis compacta* in Santa Catarina Ixtepeji, Oaxaca. Deforestation is significant, and the current climatic conditions appear to be displaced to higher altitudes. Furthermore, this epiphyte is subjected to extraction. Because of harvesting, habitat fragmentation, and climate change, we expect a declining and subdivided population of *Catopsis* in Santa Catarina with low genetic diversity, inbreeding, and declining population size. Our objectives were as follows: (1) to assess the status of the population through demographic and population genetic analyses; (2) to identify the patterns of distribution of *C. compacta* within the forest fragments; and (3) to assess the patterns of deforestation in the vegetation that host *C. compacta* using satellite images and GIS, and use this information to explain the demographic and genetic results obtained in (1).

## Materials and Methods

### The study system

**Figure 1:**
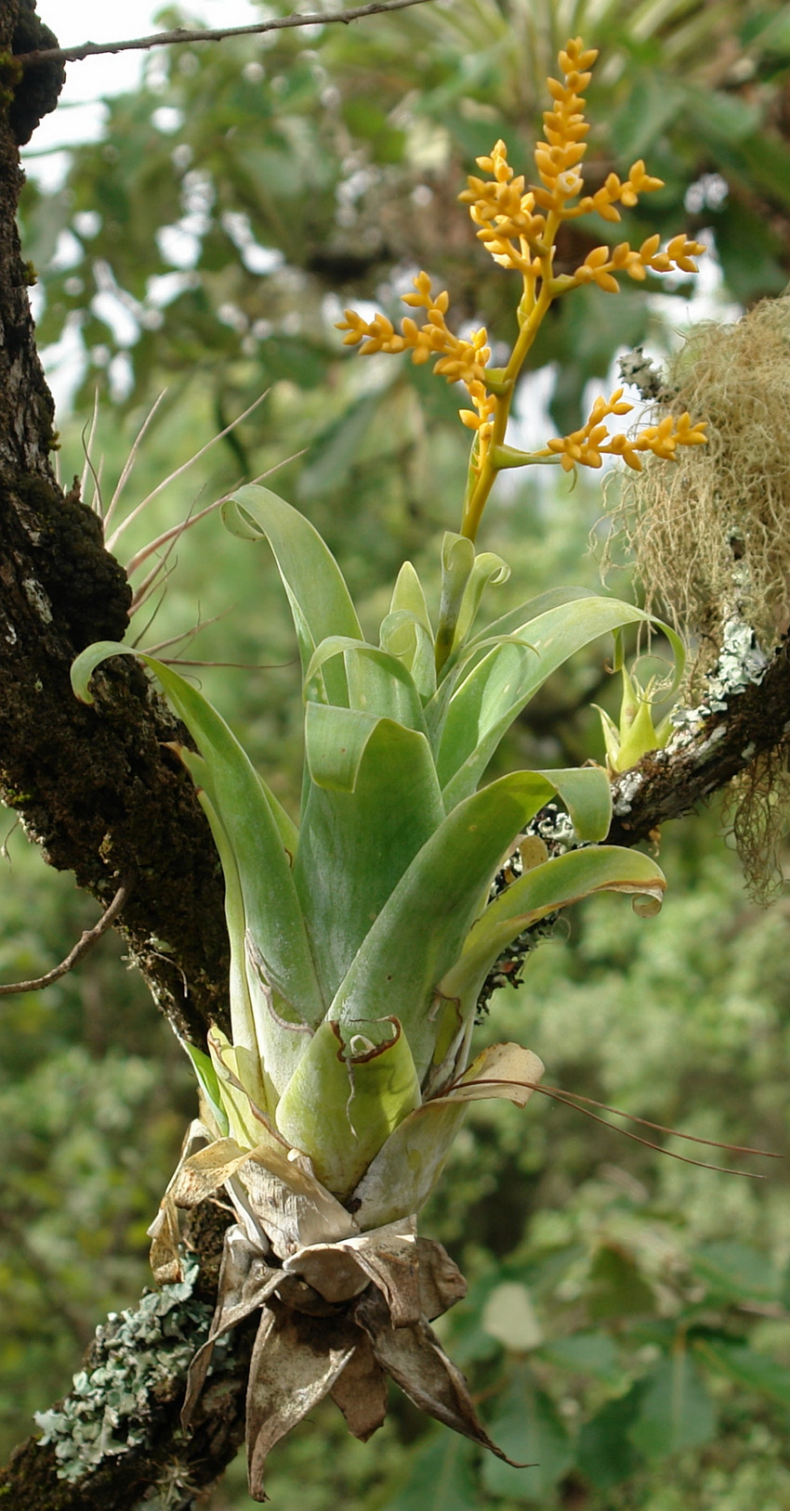
*Catopsis compacta* in its natural habitat.

*Catopsis compacta* (Schult. & Schult. F.) Mez. is a herbaceous epiphytic bromeliad from southern Mexico (Fig. 1). The leaves are compactly arranged in a tubular, 30–60 cm tall, upright rosette. A white wax covers the leaves and makes them shine when hit by the light. The flowers are bee-pollinated, and arranged in panicles with green-yellowish or yellow sepals and white petals. The species is dioecious, wind-dispersed and produce one or rarely two offshoots at the base of the rosette after blooming. Because of their beauty, *C. compacta* individuals are harvested during Christmas times near Santa Catarina Ixtepeji and sold in the local markets in Oaxaca City. This species has been intensively harvested for this purpose in Santa Catarina for at least 50 years. An estimate of 662.8 plants ha^−1^ was harvested in 2006 (Mondragón and Villa 2008).

**Figure 2:**
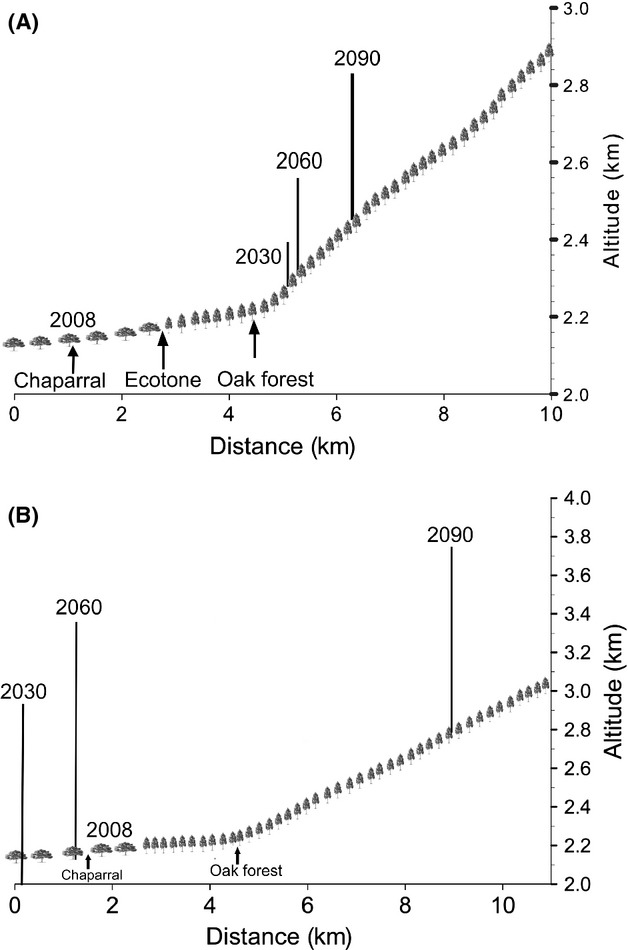
Diagrammatic representation of the ENE elevation gradient of the environs of Santa Catarina Ixtepeji, Sierra Norte, Oaxaca, Mexico showing the 2008 distribution of oak-chaparral and oak forest, the position of the study sites (arrows), and the expected range of altitudes at which the current annual temperature (A) and annual rainfall (B) of the chaparral study site will be located in 2030, 2060, and 2090 (vertical lines). Temperature and precipitation projections were obtained from the Moscow Forestry Sciences Laboratory, based on three general circulation models: GCM3 (Canadian Center for Climate Modeling, A1, A2, and B1 scenarios), HadCM3 (Hadley Centre, A2 and B1 scenarios), and GFDL (Geophysical Fluid Dynamics Laboratory, A2 and B2 scenarios, Saenz-Romero et al. 2010). The elevation range projections were calculated from linear regressions between altitude and the mean annual temperature or mean total annual precipitation reported in weather stations in Sierra Norte (Zacarías-Eslava and del Castillo 2010), and the maximum and minimum annual average temperature or total annual precipitation obtained from the projections for each projected year. Descriptions and explanations of the scenarios are available from the Intergovernmental Panel on Climate Change Data Distribution Center (http://www.ipcc-data.org/).

Two other factors may severely affect this population. Firstly, the deforestation rate is high in Santa Catarina (this study). Secondly, climate change projections obtained from the Moscow Forestry Science Laboratory predict a warming trend over the twenty-first century in this region. Rainfall projections are more variable but most of them predict a drier climate. Hence, the actual temperature and rainfall values are likely to be displaced at higher altitudes based on temperature, or rainfall versus altitude regressions. The oak forest surrounding Santa Catarina is likely to be displaced by the adjacent oak-chaparral characterized by lower height and cover and located at a lower elevation than the oak forests (Fig. 2). Both kinds of vegetation show little evidence of regeneration (Zacarías-Eslava and del Castillo 2010). Therefore, the number and size of the *C. compacta* host trees is expected to decline.

### The study site

We conducted the field work in the environs of Santa Catarina Ixtepeji, Oaxaca, where *C. compacta* is regularly harvested. The dominant vegetation near this town is a seasonal dry oak forest with *Quercus obtusata* Humb. & Bonpl., *Q. glaucoides* M. Martens & Galeotti, *Q. castanea* Née*, Q. liebmannii* Oerst. & Trel., and *Arctostaphylos pungens* Kunth as the most common species. Toward the ENE, the altitude gradually decreases from 2300 to 2140 m (Fig. 2). The vegetation becomes sparser and shorter and eventually becomes an oak-chaparral most of which plants are shrubs or short trees, dominated by *Q. glaucoides, Q. obtusata, Fuchsia macrophylla* Kunth, *Lantana hispida* Kunth, and *Dodonaea viscosa* Jacq (Zacarías-Eslava and del Castillo 2010). In addition to *C. compacta*, epiphytic species in these vegetation types include a wide variety of orchids, ferns, and other species of bromeliads: *Tillandsia bourgaei* Baker, *T. calothyrsus* Mez, *T. juncea* (Ruíz & Pav.) Poir., *T. magnusiana* Wittm., *T. prodigiosa* (Lem.) Baker, *T. usneoides* (L.) L., and *Viridantha plumosa* (Baker) Espejo (Mondragón et al. 2006). The mean annual temperature and precipitation in Santa Catarina is 18.3°C and 772.8 mm (Servicio Meteorológico Nacional 2009).

### Demography

We selected 57 host trees in the Santa Catarina oak forest. The height, the number of capsules, and the number of offshoots per plant were recorded during February 2005, 2006, 2007, and 2008. Except for few inaccessible plants, we labeled all *C. compacta* individuals established on those trees, totaling 498 individuals. The survival of these individuals and the presence of new seedlings were recorded to calculate the stage-specific survival, growth, and reproduction.

We assigned each plant to one of five categories according to its size: seedling (*s*), <1.0 cm; infantile (*i*) 1.1–2.5 cm; juvenile 1 (*j*_1_) 2.6–8.0 cm; juvenile 2 (*j*_2_) 8.1–16.0 cm; and adult (*a*) >16.0 cm, in which only female plants were considered. In *C. compacta,* the offshoots bloom only once; the new shoot produced after blooming replaces the old shoot. Therefore, we considered this kind of dynamics as stasis. As nonadult plants cannot be sexed, we assumed that the vital rates for such individuals were the same for both sexes. We built three Lefkovich projection matrices, one for each of the growth periods analyzed (2005–2006, 2006–2007, and 2007–2008). Matrices were run using MATLAB. Stochastic λ-values were generated assuming equal probabilities of occurrence of the evaluated periods, from which a mean λ-value and 95% CI were estimated.

We conducted an elasticity analysis to investigate how λ is affected by changes in vital rates caused by temporal variations (Caswell 2001). For this purpose, we focused on vital rates as opposed to matrix elements, to separate the independent effects of each vital rate on population growth (Franco and Silvertown 2004). We calculated elasticities as the proportional change in λ due to a proportional change in a vital rate as: (*x*/λ)*(δλ/δ*x*) = (*x*/λ)Σ_*ij*_((δλ/δ*a*_*ij*_)*(δ*a*_*ij*_/δ*x*)), where *x* is the vital rate (survival, growth, seed production) and *a*_*ij*_ is the transition matrix element from stage *i* to stage *j*. Unlike transition elasticities, vital rate elasticities do not necessarily sum to 1 and can be negative or positive (Caswell 2001). All demographic analyses were carried out using the popbio package in R 2.7.0 (Stubben and Milligan 2007).

With the purpose of estimating harvesting and climate change effects on the population dynamics of *C. compacta*, we developed a demographic population viability analysis, PVA (sensu Morris and Doak 2002). We estimated the probabilities of quasi-extinction of the population based on nine scenarios resulting from the combination of three harvesting and three climate scenarios. We chose three harvesting intensities: no harvesting; 10% harvesting, which approximately mimics the current harvesting rates at the study site, and overharvesting (20%). To simulate climate change, we based our analysis on water availability. Precipitation, rather than temperature, appears to be the most important climatic factor in the demography of vascular epiphytes, affecting germination, survival, and growth (Benzing 1990; Mondragón et al. 2004, in press; Zotz and Schmidt 2006). Our most humid studied period (2005–2006) was above 1 SD above the mean rainfall reported at Santa Catarina, based on 50 years records (unpublished data, National Water Commission, CONAGUA, archives). As climate change projections predict a decrease in precipitation, we simulate three scenarios of probabilities of occurrence of the λ-value associated with this period: equal probability, as observed during our 4-year observations, 0.20 and 0.02. We evaluated the probability of extinction of the *C. compacta* population in a 100-year period. Following Valverde et al. (2004), a population was considered extinct when its numbers dropped below 5% of its initial population size, assuming an initial density of 498 individuals, the sample size of this demographic study.

### Population genetic analyses

Our samples for population genetic analyses were collected in the Santa Catarina Ixtepeji forests, as in the demographic analysis. We differentiate the area in the two vegetation types described above, the oak forest and the oak-chaparral. Fresh leaf samples were collected from 71 and 63 randomly selected individuals of the oak forest and the chaparral, respectively. The leaves were extracted within 24 h after collecting and ground with liquid N_2_. The proteins were extracted with the study described by Soltis et al. (1983) extraction buffer. Horizontal starch gel electrophoresis was performed on 17 isozymes of which 11 could be scored and interpreted (Table 1). We used the pH 5.7 histidine/citrate buffer of Cheliak and Pitel (1984), and the pH 8.3 Tris-citrate/lithium borate buffer of Conkle et al. (1982) in 12.5% and 12.8% starch gels, respectively. Gels run between 3–5 h at 50 mA. We analyzed the percentage of polymorphic loci, the mean number of alleles per loci, allelic richness, and observed and expected heterozygosity under Hardy–Weinberg assumptions using the FSTAT program (Goudet 2001). Theory predicts that a bottleneck in populations under mutation-drift equilibrium causes both heterozygosity and allelic diversity to decline compared with the original population before the bottleneck. However, in recent bottlenecks of sufficient magnitude, allelic richness declines faster than heterozygosity. We looked for such transient heterozygosity excess relative to the current levels of allelic diversity with the Cornuet and Luikart (1996) bottleneck software (ver. 1.2.02). We run 1000 bootstrap permutations at 95% significance, under the infinite allele mutation model, which best fits to isozymes.

**Table 1:** Enzymes, enzyme commission number (EC No.), number of isozymes observed, stain protocol and electrode buffer used in the electrophoretic analyses of *Catopsis compacta*.

Enzymes	EC No	Number of isozymes	Electrode buffer	Stain protocol
Phosphogluconate dehydrogenase	1.1.1.44	3	Histidine	Cheliak and Pitel (1984)
Phosphoglucomutase	5.4.2.2	1	Histidine	Kress et al. (1990)
Peroxidase	11.1.7	2	Histidine	Murphy et al. (1996)
Aspartate aminotransferase	2.6.1.1	3	Lithium borate	Shaw and Prasad (1970)
Glucose 6 phosphateisomerase	5.3.1.9	2	Lithium borate	González (2004)

### Spatial distribution of the individuals and deforestation patterns

With the aid of GPS's, a team of 4–5 people recorded the position of the host individuals of *C. compacta* in the forests and shrublands of Santa Catarina through parallel walks starting at the edge of the vegetation. The trend in deforestation was analyzed through the classification of 1979 Landsat MSS and 2006 Landsat ETM+ images, spanning 27 years. The 2006 Landsat images were geometrically, radiometrically, and topographically corrected. The 1979 image was classified using the same control sites from the 2006 image and were geometrically corrected with the aid of aerial orthophotographs from 1970 of the study region. In addition to field points, the information was completed with field walks and high-resolution 2008 Google Earth images, which were georeferenced with ArcGis 9.2 and adjusted to the Landsat images. The 2006 Landsat image was classified with the maximum-likelihood method. The classification was verified with independent control points and grouped in areas with and without vegetation. The position of the host individuals was included in the 2006 Landsat image. We generated maps of vegetation cover for 1979 and 2006, and use FRAGSTAT (version 3.2, McGarigal et al. 2002) to compute the area (ha) and perimeter (km) of the vegetation patch. The rates of change in these metrics, *R*, during 1979–2006 were calculated as follows:


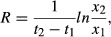


where *x*_1_ and *x*_2_ are the area or the perimeter of the patch in 1979 (*t*_1_) and in 2006 (*t*_2_) (after Puyravaud 2003).

## Results

### Demography

**Figure 3:**
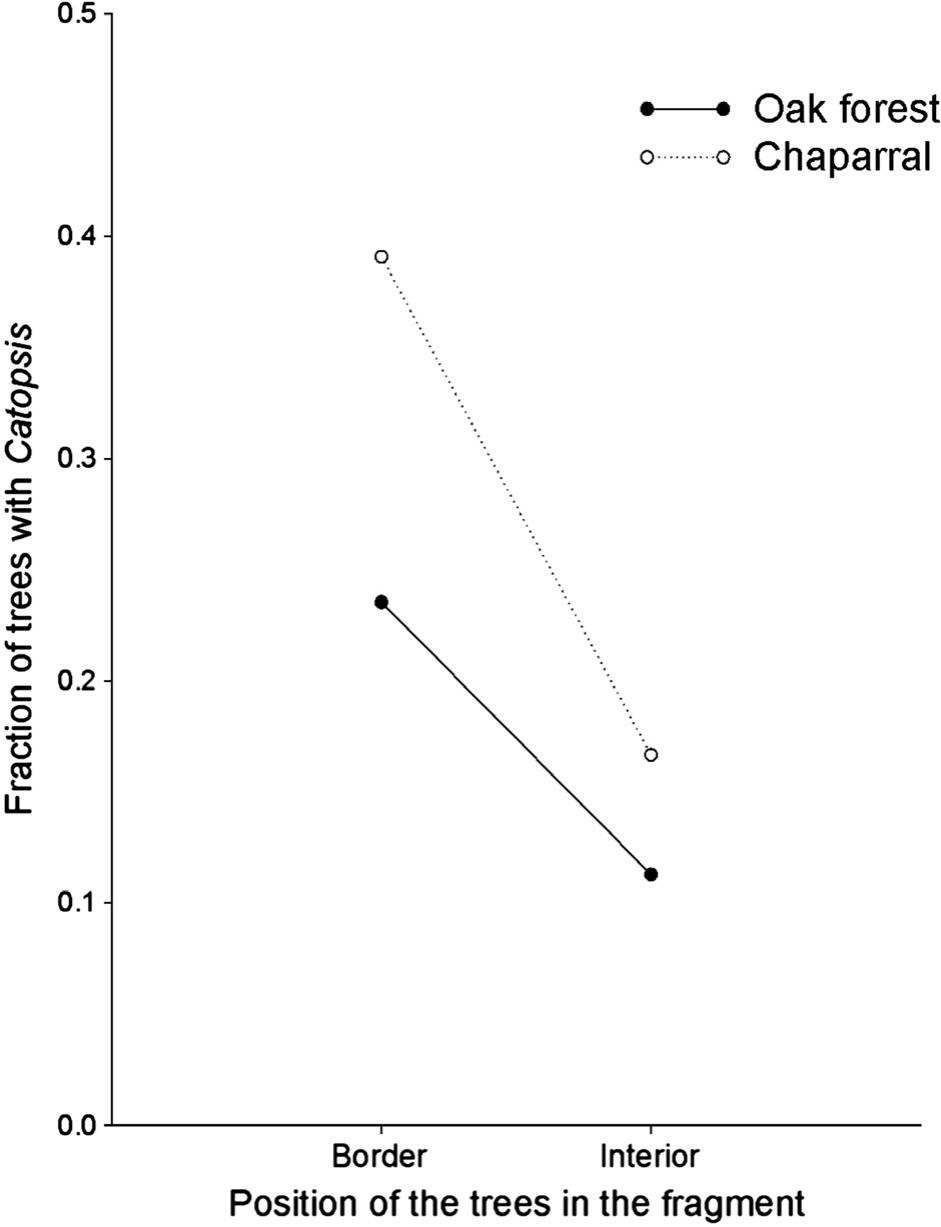
Fraction of trees in the border and the interior of chaparral and oak forest fragments in the environs of Santa Catarina Ixtepeji with at least one individual of *C. compacta*.

**Figure 4:**
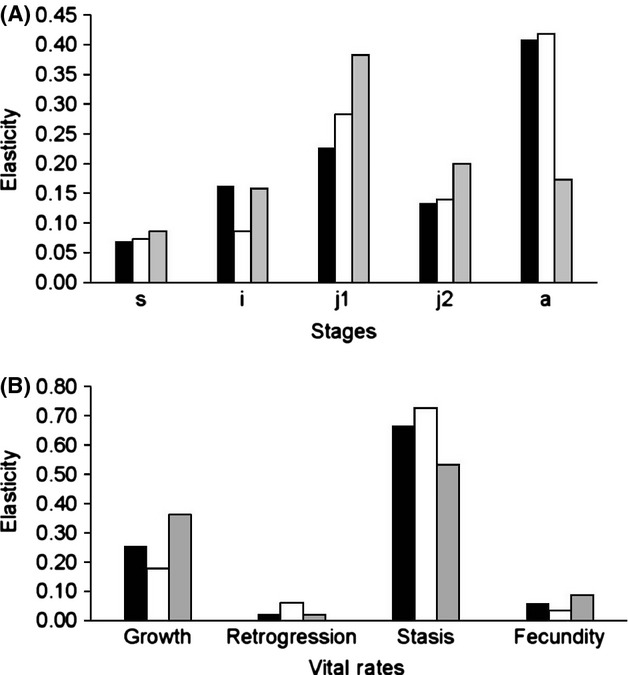
Added elasticity values (A) by category and (B) by vital rate obtained in the Santa Catarina population of *Catopsis compacta*, 2005–2006 (black), 2006–2007 (white), 2007–2008 (gray). Seedling (*s*), <1 cm; infantile (*i*) 1.1–2.5 cm; juvenile 1 (*j*_1_) 2.6–8.0 cm; juvenile 2 (*j*_2_) 8.1–16.0 cm; and adult (*a*) >16.0 cm.

**Figure 5:**
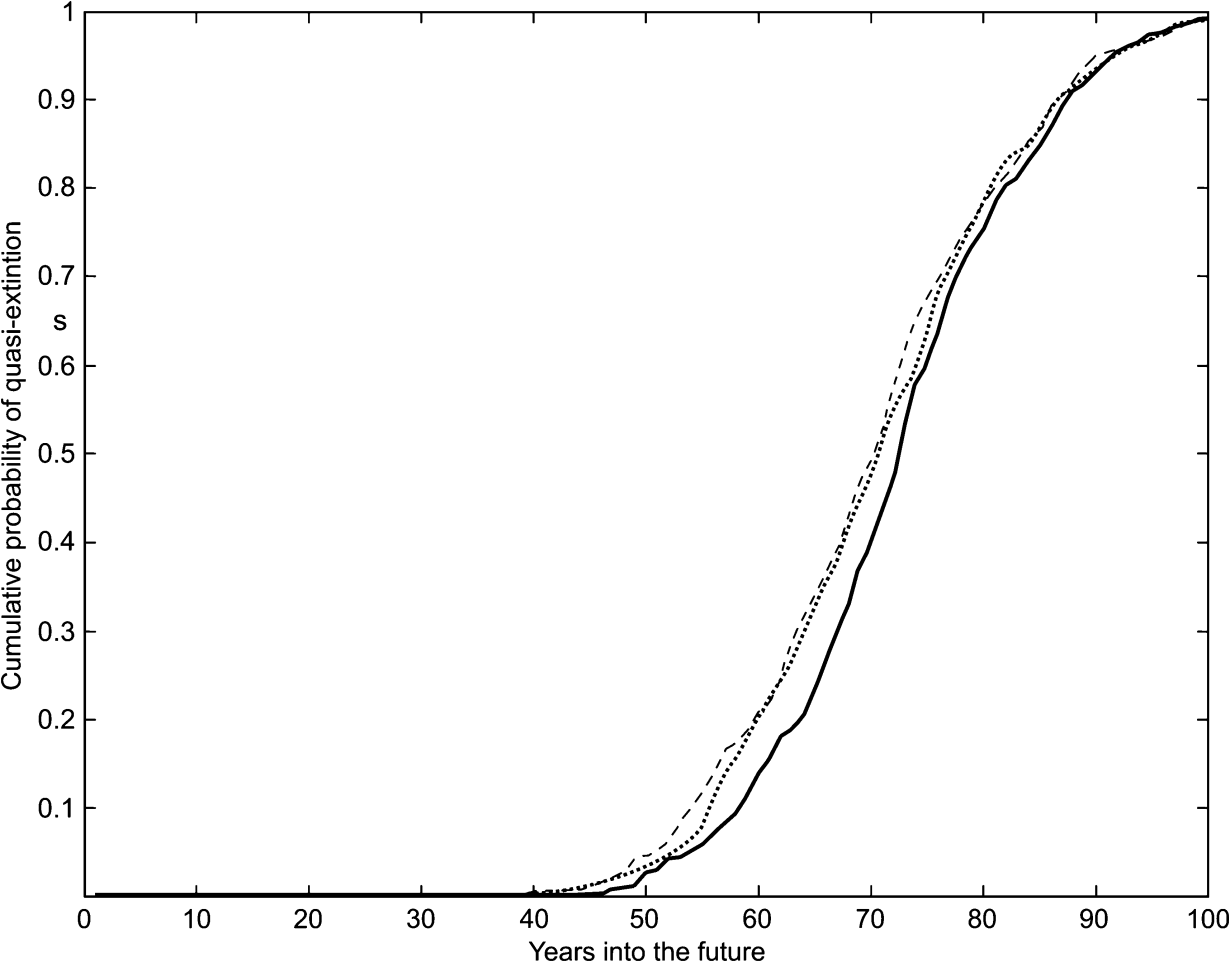
Probabilities of quasi-extinction of a *Catopsis compacta* population in Santa Catarina during a 100-year period, using 2005–2006, 2006–2007, and 2007–2008 annual matrices, with 0.333 (solid line), 0.20 (dotted line) and 0.02 (dashed line) probability of occurrence of the λ-value found in wettest year (2005) under a 20% harvesting regimen. Please note that, with 10% and 0% harvesting, all climate scenarios give zero probabilities of quasi-extinction, that is, these six scenarios are located on the *x* axis of the graph (see text for details).

When the temporal variability in demographic behavior was incorporated, the mean stochastic population growth rate, λ, of *C. compacta* was 1.047 (95% CI 1.045, 1.048), giving an intrinsic rate of increase *r* = 0.046. This λ-value estimates the expected asymptotic growth rate of the population considering the year to year oscillations detected. Thus, the population eventually will be growing at this rate if the population parameters remain constant. It is likely that the actual population growth rate of the population is close to this figure. We did not find evidence that the observed population structures in 2006, 2007, and 2008 deviate significantly from the predicted population structure by the 2005–2006, 2006–2007, and 2007–2008 matrices, respectively, either from their first iteration (χ^2^_4_ ≤ 0.44, *P* ≥ 0.98), or from the asymptotic population structure (χ^2^_4_ ≤ 0.46, *P* ≥ 0.96) (see Appendix A1). The population has not colonized all the potential hosts (Figure 3). Therefore, it is unlikely that it has approached its carrying capacity. The elasticity matrices showed that the λ-value was more dependent on the destiny of the juvenile 1 and the adult stages (Figure 4a), while the demographic processes that have the strongest effect on λ-value are stasis and growth (Figure 4b). Harvesting appears to have a larger influence on *C. compacta* demography than the increase in drought predicted by climate change. The quasi-extinction probabilities are zero without harvesting or with a harvest intensity of 10% in all the climate scenarios analyzed. However, when harvest intensity increases to 20% the quasi-extinction probabilities increased sharply after 40 years (Figure 5).

### Population genetic analyses

We could interpret and score 11 loci and 36 alleles from five enzymatic systems in the entire population analyzed. We did not find evidence of population subdivision between the fragments of the oak forest and the chaparral, as the *Fst* estimate (θ) was not significantly different from the Hardy–Weinberg expectation based on the Weir and Cockerham *F*-statistics analysis for multiple loci (θ = 0.023, 95% CI: -0.003, 0.048). Based on 900 randomizations and adjusted to nine multiple tests at 5% nominal level of significance, we could detect that seven of the nine polymorphic isozymes (77.8%) did not deviate significantly from the Hardy–Weinberg expectation. Only one of the nine polymorphic enzymes found, AAT-3, showed a significant deficit of heterozygosity, whereas PGM-1 showed a significant excess of heterozygosity. However, the multilocus *Fit* estimate revealed a significant deficit of heterozygosity (0.272, 95% CI: 0.044, 0.479), most likely attributed to a significant deficit of heterozyogosity detected at the level of individuals between subpopulations, *Fis* (0.254, 95% CI: 0.033. 0.462). All the standard measures of genetic diversity were high compared with those reported for other species of Bromeliaceae (Table 2). The Cornuet and Luikart test shows that the population is at mutation-drift equilibrium and, therefore, do not provide evidence of a recent bottleneck.

**Table 2:** Estimates of genetic diversity of *C. compacta* and other species of Bromeliaceae using isozymes. *N,* sample size; *P*, percent of polymorphic loci; *Ap*, number of alleles per polymorphic locus; *He,* expected heterozygosity.

Species	*N*	*P*	*Ap*	*He*
*Catopsis compacta* (this study)	143	81.8	3.8	0.33
*Aechmea magdalenae* (Murawski and Hamrick 1990)	47–50	33	1.8	0.10
*Aechmea tuitensis* (Izquierdo and Piñero 2000)	109	78.0	2.4	0.14
*Pitcairnia geyskesii* (Sarthou et al. 2001)	162	63.3	1.9	0.18
*Tillandsia achyrostachys* var. *achyrostachys* (González-Astorga et al. 2004)	–	59.4	1.9	0.13
*Tillandsia ionantha* (Soltis et al. 1987)	243	16.7	1.4	0.07
*Tillandsia recurvata* (Soltis et al.^1987^)	–	3.8	1.0	0.01

### Spatial distribution and plant cover dynamics

A categorical analysis of variance using proc CATMOD of SAS 9.1 revealed that: (1) trees colonized by at least one individual of *C. compacta* are more common in the chaparral than in the oak forest (χ^2^_1_ = 7.69, *P* = 0.0055); and (2) in both kinds of vegetation, trees at the border of the fragments are more likely to be colonized by *C. compacta* than trees at the interior of the fragments (χ^2^_1_ = 7.69, *P* = 0.0004) (Fig. 3). Based on GIS analysis, the border of the fragment was defined by trees in which at least 50% of its canopy horizontal projection is separated from the canopy horizontal projection of other trees by at least 7 m, which is the average canopy diameter of the trees. Otherwise, the trees were considered as located in the interior of the fragment. The analysis of deforestation revealed that the oak forest and oak-chaparral decreased at an annual rate of 5.09 and 5.60%, respectively, while the perimeter of border of the these two vegetation types increased at an annual rate of 4.70 and 3.43%, respectively, based on the analyses of satellite imagery spanning 27 years (1979–2006).

## Discussion

Species inhabiting human-altered environments are commonly affected by a combination of potential threatening factors of anthropogenic origin, such as harvesting, deforestation, and climate change, which may jeopardize the persistence of their populations. Harvesting is predicted to modify the structure and dynamics of the populations, alter population subdivision, decrease genetic diversity, and induce selective genetic changes (Allendorf et al. 2008). When the entire individual is removed, as is the case of *C. compacta*, harvesting imposes an additional source of mortality, because the harvested individual no longer contributes offspring and genes to the population. Habitat fragmentation, a widespread consequence of perturbation of natural habitats, is commonly expected to cause habitat losses and increases in the degree of isolation of the remaining populations (Lindenmayer and Fischer 2006). Habitat losses or at least habitat displacements, due to changes of the precipitation and temperature regimens are also an expected consequence of climate change (Wilson and Gutiérrez 2012). Habitat losses, mortality, population, isolation, and reductions in population genetic diversity, in turn, are likely to increase the probabilities of extinction by genetic and demographic stochasticty (Caswell 2001; Allendorf and Luikart 2007). As such, the net impact of climate change, fragmentation and harvesting can be expected to be even harsher than the separate impacts of each of these factors, because all of them can stress individually the populations. The present study, however, shows that *C. compacta* can maintain a growing population and displays a high genetic variation, even with harvesting and high levels of fragmentation and provides evidence that antagonistic effects of fragmentation with harvesting can explain, at least in part, these results.

In small or subdivided populations, such as those affected by harvesting and habitat fragmentation, genetic drift is expected to reduce population genetic diversity (Allendorf and Luikart 2007). Indeed, according to a recent meta-analysis, habitat fragmentation decreases population genetic diversity in most studied species (Aguilar et al. 2008), and harvesting may do so in some cases (e.g., Cruse-Sanders and Hamrick 2004). Ideally, assessing the effects on potential stressors of the populations requires comparisons of several populations before and after the effects of such stressors take place. In lieu of such kind of comparison, testing for population subdivision, and looking for evidences of past bottlenecks can help to reveal the expected effects of such stressors on the genetic attributes of the populations. The reported genetic variation of other species with similar breeding systems, dispersal mechanisms, or taxonomic affinity can provide a surrogate reference for comparative purposes with the genetic variation observed in the tested population. For this purpose, the use of allozymes as genetic markers results convenient given the extensive number of species studied and published meta-analysis, such as that of Hamrick and Godt (1996) with 247 plant species analyzed (but see Lowe et al. 2004, for some drawbacks of this kind of meta-analysis).

In spite of at least 50 years of harvesting and a continuous fragmentation of the habitat for at least 27 years, the population of *C. compacta* does not show evidences of population subdivision or past bottlenecks. The significant *Fis* detected in our multilocus analysis is most likely due to mating among relatives particularly between individuals sharing the same host trees as pollinators tend to fly short distances (personal observation). On the other hand, the nonsignificant *Fst* value suggests that the current trends of fragmentation have not caused population subdivisions between the oak forest and the chaparral fragments. The estimators of genetic diversity found in *C. compacta* were the highest reported in Bromeliaceae (Table 2) and are among the highest reported in plant species from other families. For instance, our values of genetic diversity (0.405 vs. 0.157, outcrossing-wind-dispersed seeds; or 0.165, outcrossing-monocots) and percentage of polymorphic loci (81.8 vs. 62.4%, outcrossing-wind-dispersed seeds; or 52.5% outcrossing-monocots) were higher than the average reported in species with similar breeding system and seed dispersal mechanism, or taxonomic group using isozymes (cf., Hamrick and Godt 1996). In summary, our data do not show evidences that genetic erosion, population subdivision between adjacent vegetation fragments, or past bottlenecks have affected the population of *C. compacta*.

The high genetic variation in *C. compacta* requires further investigations, but at least five factors may contribute to this result. Firstly, dioecy prevents selfing, the most extreme source of inbreeding. Secondly, among wind-dispersed propagules, plumed seeds, such as those *C. compacta*, are reportedly highly effective for long-distance dispersal (Cousens et al. 2008). Thirdly, the epiphytic condition may favor long-distance dispersal, by positioning the plants on the host trees, from which height dispersal can reach longer distances than ground positions. Fourthly, as explained below, the expansion of the perimeter of the fragments, during vegetation fragmentation, as shown by the GIS analyses, may have favored long-distance seed dispersal. Gene flow reduces inbreeding, decreases the losses of genetic variation by drift, and homogenizes subpopulations as supported by the nonsignificant *Fst* value. Finally, the expansion of the *C. compacta* population, as suggested by the demographic analyses, may contribute to decrease the probabilities of losses of genetic variation by drift, and to generate genetic variation by mutations.

Fragmentation is not a synonymous of habitat loss, but a change in landscape structure (Fahrig 2003). One of the most obvious changes in structure during fragmentation is the formation of borders in the vegetation fragments, which are interfaces between the fragments and the habitat that surrounds them. In Santa Catarina, the total area of the forest decreased 76% on average in 27 years. However, our plant distribution analysis shows that most *C. compacta* individuals grow on host plants located in a fringe of vegetation at the border of the fragments. The fragment perimeter increased at a relatively high rate in both the oak forest (0.047 years^−1^, *t*_*d*_ = 14.8 years) and the oak-chaparral fragments (0.034 years^−1^, *t*_*d*_ = 20.2 years), displaying similar values to the estimated intrinsic rate of increase in the population (0.046 years^−1^, *t*_*d*_ = 15.1 years). Logging is a factor that may have contributed to increase open spaces in the vegetation. Wood is commonly extracted for diverse purposes including firewood and lumber. Climate change is another factor. A drier and warmer climate trend is likely affecting the study area. Under such conditions, oak forests are expected to be replaced by oak-chaparrals with low height and low canopy cover (Fig. 2). The greater abundance of open spaces in the chaparral may have increased the probabilities of *C. compacta* colonization (see Fig. 3). Other species of epiphytes appear to be benefited by open spaces such as those with water-absorbing trichomes, the atmospheric-type epiphytes, which usually replace more mesic species under such conditions (e.g., Flores-Palacios and García-Franco 2004; Winkler et al. 2005; Cascante-Marín et al. 2006; Werner 2011).

In *C. compacta*, the edges of the vegetation may result more convenient than the forest interior for both physiological and genetic reasons. Firstly, *C. compacta* has thick leaves with a thick cuticle and water-absorbing trichomes, which are well-recognized adaptations to xeric environments in Bromeliaceae (Medina 1974). Trichomes, however, partially block the incidence of light to the chloroplasts and hinder gas-exchange reducing, therefore, photosynthetic rates, particularly in shady environments. The oak forests near Santa Catarina have a relatively dense foliage (73% cover; Zacarías-Eslava and del Castillo 2010), which may reduce the probabilities that *C. compacta* individuals in the core of fragments display a positive carbon balance, particularly during the rainy season, when the host trees are fully covered by leaves. By contrast, the plants on hosts at the edge of the fragments are expected to receive more lateral light on the open side of the edges, increasing the probabilities that xeromorphic epiphytes, such as *C. compacta,* display a positive carbon balance. This conclusion is also supported by the fact that our PVA analyses show that quasi extinction probabilities are zero even when the frequency of rainy years decrease to 2%. Secondly, open spaces favor a laminar flow of the wind, compared to spaces with complex vegetation, which promotes turbulence (Whitehead 1983). Thus, for species with wind-dispersed seeds, the edge of the fragments likely increase gene flow and decrease population subdivision caused by habitat fragmentation by favoring long-distance dispersal. Gene flow appears to be favored by fragmentation in other plant species (see Young and Clarke 2000, for some examples).

The increases in the perimeter of the vegetation patches and the openness of the vegetation, likely enhanced by climate change and logging, appear to favor the population expansion of *C. compacta* in Santa Catarina, by increasing the habitat at which new individuals can become established and by favoring long-distance seed dispersal. This result and the fact that all possible hosts have not been colonized indicate that habitat size is not constraining the population growth of *Catopsis,* under the prevailing conditions. By contrast, harvesting increases the mortality of the adults, one of the stages that most contributes to population growth, according to our elasticity analysis. Harvesting was also the factor with the highest influence in quasi-extinction probabilities according to our PVA. Climate change and deforestation appear to increase the habitat and the opportunities for higher dispersal of *Catopsis*, but harvesting partially counteracts such effects by reducing the number of reproductive individuals. At present, however, the levels of harvesting do not appear to harm significantly the population, which appears to keep growing, maintain a stable size structure and a high genetic diversity.

The conditions on which our analyses based are likely to change, and the prevalence of this species in the future in Santa Catarina is not warranted. If the levels of harvesting duplicates, the risks of extinction increases sharply, as indicated by our PVA. In general, the total edge of the fragments tends to increase fast during the early stages of fragmentation. As fragmentation proceeds, the rate of increase of the perimeter is expected to decline, and, eventually, the total edge decreases first slow and then fast had fragmentation continue (Fahrig 2003). Indeed, in the less fragmented oak forest, the rate of increase in the perimeter was higher than that in the more open oak-chaparral. If the estimated rates of deforestation continue, the perimeter of the vegetation patches are likely to decline, together with the extinction risks of *C. compacta* owing to the ecological and genetic stochasticity. Eventually, the distribution of *C. compacta* is expected to be positively correlated with vegetation as predicted in simulations (Hsu et al. 2012).

Traditionally, the creation of reserves has been identified as the ultimate solution for biodiversity conservation. For species such as *C. compacta*, however, undisturbed reserves are not an option. These species requires periodic disturbances that create forest openings. We have shown that *C. compacta* can take advantage of moderate levels of disturbance and a warmer and drier climate both of which may generate open spaces in the vegetation on whose borders this species can prosper. For edge-inhabiting species, the creation of artificial edges appears to be a good prescription for conservation and management (Lindenmayer and Fischer 2006). Moderate plant extraction and moderate logging may create artificial edges in Santa Catarina bringing about benefits to the local people. However, a further complication arises, because *C. compacta* and their hosts have contrasting requirements for survival and reproduction, as suggested by the fact that *C. compacta* population has a positive and significant stochastic λ-value but the forest is decreasing and regeneration is poor (Zacarías-Eslava and del Castillo 2010). Conservation and management practices should take a careful consideration of the requirements of both the host and the epiphytes together with the dynamics of the habitat, and the demographic and genetic context of the species to be preserved. Certainly, a close monitoring at population and community level is needed in combination with adaptive management techniques (Millar et al. 2007). These practices should be particularly relevant for species inhabiting transient habitats such as those affected by climate change and disturbance for a sound management and conservation planning.

*Catopsis compacta* is an example of a species in which different anthropogenic activities, such as harvesting and deforestation, probably induced by logging and climate change, can inflict antagonistic effects on the population under certain conditions, and display contrasting responses to such factors relative to its hosts. The conservation and sustainable management of this kind of system require a clever administration of disturbance rather than preventing it through reserves in which disturbance is suppressed. A full understanding of the consequences of potential threatening factors on species persistence or extinction should only be accomplished by inspecting the action and interaction of all factors that might affect the population and with the consideration of such effects on the species on which the focus species depends. For these goals, our results illustrate the advantages of multidisciplinary approaches, each of which provide relevant and essential information.

## Appendix A1

**Transition matrices and main demographic results for the *C. compacta* population studied from 2005 to 2009 in Santa Catarina Ixtepeji. λ-values and confidence intervals (95%). *S,* seedling; *I,* infantile; *j*_1,_ juvenile 1; *j*_2_, juvenile 2; *a,* adult, *w,* stable size distribution; *v*, size-specific reproductive values.**

**Table:**

Transition matrix								
			
	*s*	*i*	*j*_1_	*j*_2_	*a*	Observed structure	*w*	*v*
2005–2006								
λ = 1.061 (1.006, 1.113)								
*s*	0.167	0.000	0.000	0.000	2.350	0.014	0.189	1.000
*i*	0.750	0.622	0.081	0.000	0.000	0.267	0.378	1.193
*j*_1_	0.000	0.250	0.721	0.137	0.000	0.534	0.302	2.099
*j*_2_	0.000	0.000	0.093	0.510	0.067	0.130	0.060	6.638
*a*	0.000	0.000	0.000	0.235	0.867	0.054	0.072	14.338
2006–2007								
λ = 0.935 (0.847, 1.025)								
*s*	0.500	0.000	0.000	0.000	0.330	0.010	0.171	1.000
*i*	0.250	0.392	0.061	0.000	0.000	0.164	0.116	1.742
*j*_1_	0.000	0.459	0.696	0.167	0.000	0.645	0.331	2.061
*j*_2_	0.000	0.000	0.101	0.528	0.133	0.132	0.156	2.516
*a*	0.000	0.000	0.027	0.139	0.800	0.049	0.226	4.912
2007–2008								
λ = 1.056 (0.836, 1.137)								
*s*	0.333	0.000	0.000	0.000	8.000	0.012	0.237	1.000
*i*	0.667	0.404	0.049	0.000	0.000	0.139	0.272	1.083
*j*_1_	0.000	0.404	0.768	0.053	0.000	0.639	0.395	1.746
*j*_2_	0.000	0.000	0.092	0.553	0.071	0.155	0.075	4.900
*a*	0.000	0.000	0.000	0.158	0.500	0.055	0.021	15.027
